# Computational-experimental strategy identifies Co-upregulated biomarkers linking coronary heart disease and type 2 diabetes pathogenesis

**DOI:** 10.3389/fgene.2025.1673303

**Published:** 2025-11-12

**Authors:** Wei Li, Yu Cao, Chen Yu, Bingjun Che, Miao He, Dongbiao Li, Lihong Jiang, Lijing Ma

**Affiliations:** 1 Department of Cardiovascular Surgery, The First People’s Hospital of Yunnan Province, The Affiliated Hospital of Kunming University of Science and Technology, Kunming, China; 2 Department of Neurosurgery, The First Affiliated Hospital of Kunming Medical University, Kunming, China; 3 Medical College, Yunnan College of Business Management, Kunming, China; 4 Faculty of Life Science and Technology, Kunming University of Science and Technology, Kunming, China; 5 Department of Endocrinology, National Clinical Key Specialty Cultivation Project Platform for Endocrinology, The First People’s Hospital of Yunnan Province, The Affiliated Hospital of Kunming University of Science and Technology, Kunming, China; 6 Department of Endocrinology, NHC Key Laboratory of Healthy Birth and Birth Defect Prevention in Western China, The First People’s Hospital of Yunnan Province, The Affiliated Hospital of Kunming University of Science and Technology, Kunming, China

**Keywords:** type 2 diabetes, coronary heart disease, diagnostic biomarkers, transcription factor, bioinformatic technology

## Abstract

**Background:**

Coronary heart disease (CHD) and type 2 diabetes (T2D) represent a significant global comorbidity burden, with shared yet incompletely understood molecular mechanisms. This study aimed to identify shared diagnostic biomarkers and elucidate core pathways linking CHD and T2D pathogenesis.

**Methods:**

Integrated bioinformatics of CHD/T2D transcriptomes identified shared differentially expressed genes (DEGs) and co-expression modules via Weighted Gene Co-expression Network Analysis (WGCNA). Receiver operating characteristic (ROC) analysis selected CPD, GGCT, SUZ12, and ZMYM2 as top diagnostic biomarkers. These predictions were validated using C57BL/6 and ApoE^−/−^ mouse models of T2D/CHD. Aortic tissues underwent histopathology (Hematoxylin and Eosin (H&E), Oil Red O, Sirius Red) and multi-level molecular assays (immunofluorescence, Western blot, reverse transcription quantitative polymerase chain reaction (RT-qPCR).

**Results:**

Bioinformatics revealed 328 shared DEGs, with CPD, GGCT, SUZ12, and ZMYM2 showing high diagnostic efficacy. T2D mice exhibited persistent hyperglycemia. Aortic histopathology confirmed disease-specific changes: atherosclerotic plaques in CHD and vascular basement membrane thickening in T2D. Critically, all four biomarkers showed concurrent upregulation in diseased vessels at both protein (immunofluorescence, Western blot) and mRNA (RT-qPCR) levels.

**Conclusion:**

This study establishes CPD, GGCT, SUZ12, and ZMYM2 as shared CHD/T2D diagnostic biomarkers. Their validated co-upregulation highlights their dual-disease diagnostic and therapeutic potential.

## 1 Introduction

Coronary Heart Disease (CHD), also known as ischemic heart disease or coronary atherosclerotic heart disease, refers to heart disease caused by atherosclerosis of the coronary arteries leading to luminal stenosis or occlusion, resulting in myocardial ischemia or necrosis (Global, 2018a). Its pathological basis is abnormal lipid metabolism, where lipids in the blood deposit on the arterial intima, forming atherosclerotic plaques (appearing as white, lipid-laden lesions). As the core manifestation of cardiovascular diseases (including stroke, heart failure, peripheral arterial disease, etc.), CHD is a leading cause of global mortality (Global, 2018b), severely impairing patients’ quality of life, and constitutes a major global health burden: the 2019 Global Burden of Disease study reported approximately 197 million people affected worldwide, leading to 9.14 million deaths (Roth et al., 2020). This impact is global in scope but particularly prominent in Europe, accounting for half of all deaths there (Visseren et al., 2022). Regional data show: in the United States, the 2023 American Heart Association (AHA) Heart Disease and Stroke Statistics Update indicated approximately 20.5 million adults aged ≥20 years were affected (prevalence rate of 7.1%) (Tsao et al., 2023); in China, CHD became the second leading cause of death by 2016, exhibiting a concerning trend potentially towards becoming the primary cause (Zhao et al., 2019). Life-threatening acute coronary syndromes are primarily triggered by rupture of “vulnerable” plaques. These plaques are characterized by a thin, fragile fibrous cap (covering a large lipid-rich necrotic core with sparse vascular smooth muscle cells), extensive macrophage infiltration, and insufficient collagen repair and angiogenesis (Davies, 1995; Ber et al., 2021). Although CHD incidence rates have declined in some countries in recent years, it remains a formidable and persistent global public health challenge.

Diabetes is a chronic disease characterized by elevated blood glucose levels, resulting from insufficient insulin production by the pancreas or insulin resistance in peripheral tissues (Classification and Diagnosis of Diabetes, 2019). Type 2 diabetes (T2D) accounts for over 90% of all diabetes cases, and its prevalence is rapidly increasing worldwide. The International Diabetes Federation projects that the number of adults with diabetes will rise from 463 million in 2019 to 700 million by 2045, with the proportion of T2D cases continuing to grow in most countries (Saeedi et al., 2019). Amid rapid population aging, diabetes has emerged as a major public health concern, involving multiple pathogenic factors that accelerate aging (Palmer et al., 2015). Its complications—including heart disease, stroke, and kidney failure—are primarily driven by damage to the micro- and macrovascular beds (Roglic and Unwin, 2010). Cardiovascular disease represents the leading cause of mortality, with CHD posing a particularly severe threat to individuals with T2D (Go et al., 2014; Tsiachris et al., 2011; Siddiqui and Maddaloni, 2021). Epidemiological studies confirm that middle-aged T2D patients face a 2-to-4-fold higher CHD risk compared to non-diabetic individuals (Sattar et al., 2019). Notably, traditional CHD risk factors (hypertension, dyslipidemia, smoking) exhibit substantially amplified pathogenic effects in the diabetic population (Fuller et al., 1983; Rosengren et al., 1989). Concurrently, hyperglycemia itself directly drives coronary atherosclerosis through mechanisms including vascular endothelial inflammation activation (Pan et al., 2013), accumulation of advanced glycation end products (AGEs) (Prasad and Tiwari, 2017), and dysregulated lipid metabolism (Srivastava, 2018; Femlak et al., 2017), establishing diabetes as an independent risk factor for CHD (Clodi et al., 2023; Yang et al., 2021).

T2D patients demonstrate significant genetic susceptibility to developing subsequent cardiovascular outcomes (Lu et al., 2020; Vujkovic et al., 2020). Research has identified the GLUL locus, implicated in glutamate metabolism, as associated with elevated cardiovascular risk in diabetic individuals (Qi et al., 2013; Qi et al., 2011). Furthermore, both conditions share other genetic susceptibility loci, such as PCSK9 and RAC1 genes (Wu et al., 2024). These genetic backgrounds interact with environmental exposures and lifestyle factors within a multi-layered network that collectively mediates disease pathogenesis (Vujkovic et al., 2020). However, it should be noted that not all T2D patients develop vascular complications such as CHD. This observation does not negate the strong association between T2D and CHD; rather, it indicates that although the two diseases share critical pathological drivers, the progression to CHD in individual patients may also involve partially independent mechanisms. In this context, distinguishing between“shared mechanisms” (driving comorbidity) and “independent mechanisms” (determining individual risk) is crucial. Systematic elucidation of the shared molecular mechanisms and regulatory networks underlying T2D–CHD comorbidity provides the essential foundation for identifying synergistic therapeutic targets. Such targets would act on core pathways common to both diseases and benefit the large population of T2D patients at high risk of CHD, who carry the major burden of this comorbidity.

This study employs an integrated bioinformatics approach by acquiring independent T2D and CHD datasets from the Gene Expression Omnibus (GEO) database, applying WGCNA to identify disease-associated modules, and utilizing DEG screening to pinpoint shared genes. Subsequent functional enrichment analysis explores comorbid pathways, while ROC curve evaluation validates diagnostic efficacy of key biomarkers through experimental verification, followed by prediction of transcription factors (TFs) regulating these genes. This systematic pipeline ultimately aims to decipher the genetic interplay in T2D-CHD comorbidity to establish a molecular basis for precision intervention.

## 2 Materials and methods

### 2.1 Data source

Both CHD and T2D datasets were retrieved from the GEO database. CHD transcriptomic data originated from GSE113079 (Platform: GPL20115), comprising mRNA profiles of peripheral blood mononuclear cells (PBMCs) from 93 CHD patients and 48 healthy controls.T2D data were derived from GSE41762 (Platform: GPL6244), which includes islet tissue mRNA expression profiles of 57 non-diabetic controls and 20 T2D patients, assayed using GeneChip®Human Gene1.0ST arrays.

### 2.2 Identification of DEGs

We performed data quality control, processing, and statistical analysis using the limma R package on mRNA expression profiles from the GEO database, comparing normal samples to those with coronary heart disease and/or Type 2 diabetes (CHD/T2D). DEGs were defined as those with an adjusted *P* < 0.05 (Liu et al., 2022). Differential gene expression patterns were visualized through hierarchical clustering heatmaps and volcano plots generated using R software.

### 2.3 Characterization of modules and genes affiliated with CHD

WGCNA was performed on the CHD cohort (GSE113079) to identify phenotype-associated modules and genes. Outlier samples were filtered using the “goodSamplesGenes” function to ensure analytical robustness. We constructed a scale-free network by selecting the optimal soft-thresholding power (β), computed gene adjacency matrices, and performed hierarchical clustering. Gene modules were identified through dynamic tree-cutting (minimum module size = 100 genes) and subsequently merged at a module eigengene dissimilarity threshold of 0.2. Modules showing the highest correlation with CHD phenotype were determined via Pearson correlation. Genes within these key modules were designated CHD-associated. To validate biological relevance, we calculated Pearson correlations between Module Membership (MM) and Gene Significance (GS) for all genes.

### 2.4 Identification of shared genes and pathway enrichment

A combined analysis of the genes screened by WGCNA and DEGs was conducted by drawing Venn diagrams. Overlapping genes were considered core shared genes and were extracted for further functional enrichment analysis. We then used the “heatmap” R package to map the genes expression heatmap. Gene Ontology (GO)and Kyoto Encyclopedia of Genes and Genomes (KEGG)pathway enrichment analyses were performed using the “clusterProfiler” (Yu et al., 2012), packages in R. Statistical significance was set at *P* < 0.05.

### 2.5 The diagnostic value of candidate biomarkers

Using the pROC R package (Robin et al., 2011), we generated ROC curves for common genes in both CHD and T2D cohorts. Genes demonstrating diagnostic potential (Area Under the Curve (AUC) > 0.8) in discriminating control from disease samples were designated candidate critical common genes. Cross-tabulation identified cohort-overlapping genes, establishing the final critical common gene set.

### 2.6 Generation of a critical common gene-transcription factor (TF) network

Using Network Analyst (http://www.networkanalyst.ca), we predicted TFs regulating critical common genes under disease conditions, applying filters for intensity signal (<500) and confidence score (<1). The resulting TF-gene regulatory networks were visualized in Cytoscape. We subsequently assessed differential TF expression between control and disease states across CHD and T2D cohorts using Wilcoxon rank-sum tests (*P* < 0.05).

### 2.7 Experimental assessment

#### 2.7.1 Reagents and chemicals

Streptozocin (STZ pure: ≥98%; Chemical Abstracts Service (CAS): 18883-66-4) was purchased from Biosharp (China). 1%Sodium Citrate Buffer Solution (C1013), Oil Red O staining Kit (G1236) and Sirius Red (GP1033) were purchased from Solabio (China). Radio-Immunoprecipitation Assay (RIPA) lysis buffer (R0010) was purchased from Servicebio (China). Bicinchoninic Acid (BCA) Kit (PC0010) was purchased from Beyotime (China). Goat pAb to Rabbit (Rb) IgG H&L (ab150077) and Goat pAb to Mouse (Ms) IgG H&L (ab150113) were purchased from abcam (China).

#### 2.7.2 Animals and treatment

The C57BL/6 mice used in this study were purchased from SPF Biotechnology (Beijing, China; batch No. 110324241105933338), and the ApoE^−/−^mice were obtained from SPF Biotechnology (Beijing, China; batch No. 110324241105933683). Mice were maintained under specific pathogen-free conditions at the Kunming Medical University Experimental Animal Center. Animals were housed at a controlled temperature (22 °C ± 1 °C) and relative humidity (45%–55%) on a light/dark cycle (standard conditions), with food and water available *ad libitum*. Eight-week-old male mice were divided into three groups (n = 6 per group) (Global, 2018a): Normal control (NC): C57BL/6 mice (Global, 2018b); Type 2 diabetes (T2D): C57BL/6 mice (Roth et al., 2020); Coronary Heart Disease (CHD): ApoE^−/−^ mice.Sample size and power: An *a priori* power analysis was performed using G*Power 3. 1 for a one-way ANOVA (three groups, n = 6 per group; total N = 18), with α = 0. 05 and an anticipated effect size of Cohen’s f = 1. 03. The analysis indicated an achieved power (1 − β) of 0. 95, supporting the adequacy of the planned sample size to detect large between-group effects.

The T2D model was established in C57BL/6 mice using a modified protocol (Wang et al., 2012). Mice received a high-fat diet (HFD) for 6 weeks to induce insulin resistance, followed by a single intraperitoneal streptozotocin injection (STZ, 75 mg/kg). Diabetes was confirmed by two consecutive fasting blood glucose (FBG) measurements >11.1 mmol/L. The CHD group received a Western-type diet (21% fat, 34% sucrose, 19.5% casein, 0.2% cholesterol; Envigo) for 12 weeks. The NC group was administered sodium citrate buffer injections and maintained on standard chow diet. Body weight and blood glucose were monitored weekly in all mice throughout the 15-week experimental period (weeks 0–15). At the end of the diet protocol, the animals were humanely euthanized using 80 mg/kg ketamine–10 mg/kg xylazine. In our study, aortic arch and aortic root dissection were performed.

#### 2.7.3 Histological and immunofluorescence assessment

Mouse aortic tissues were fixed in 4% paraformaldehyde (G1119, Servicebio) for 24 h, followed by sequential dehydration through a graded ethanol series, xylene clearing, and paraffin embedding; serial 4-μm sections were cut using a paraffin microtome (RM2235, Leica), baked at 60 °C for 30 min, deparaffinized in xylene, and rehydrated prior to histological staining using H&E, Oil Red O, and Sirius Red according to manufacturers’ protocols, with subsequent quantification of total tissue area, Oil Red O-positive area, and Sirius Red-positive area performed using ImageJ software (Meng et al., 2019).

An additional set of sections underwent immunofluorescence staining using the following primary antibodies as required: GGCT (bs-13346R, Bioss, China), CPD (DF9339, Affinity, China), ZMYM2 (bs-13603R, Bioss, China), and SUZ12 (R381543, Zenbio, China); post-staining imaging was performed using a laser scanning confocal microscope (Olympus, Japan).

#### 2.7.4 Western blotting

Mouse aortic tissue was collected, and total proteins were isolated from each sample and separated on 8% Sodium Dodecyl Sulfate Polyacrylamide Gel Electrophoresis (SDS-PAGE) gels. Proteins were then transferred to Polyvinylidene Fluoride (PVDF) membranes (Millipore, Burlington, MA, United States), followed by blocking with 5% skim milk (BD, San Jose, CA, United States) at room temperature for 1 h. Membranes were incubated overnight at 4 °C with specific primary antibodies as follows: CPD (DF9339, Affinity, United States), GGCT (bs-13346R, Bioss, China), SUZ12 (R381543, Zenbio, Chengdu, China), ZMYM2 (bs-13603R, Bioss, Beijing, China), and β-Actin (#66009-1-Ig, Proteintech, China). Afterward, membranes were incubated with HRP-conjugated secondary antibodies (Servicebio, China) at room temperature for 1 h. Detection was performed using a chemiluminescence apparatus (Beijing Sage Creation, Beijing, China) with enhanced chemiluminescence (ECL, GE, Boston, MA, United States). The density of the protein bands was measured using ImageJ software.

#### 2.7.5 RT-qPCR

Total RNA extraction was performed with the Trizol method (15596-018CN, Ambion, United States), followed by reverse transcription of mRNA to cDNA. Quantification of gene transcription levels utilized SYBR Green PCR Master Mix (Thermo Fisher Scientific, MA) on a Roche Real-Time PCR system. Primer sequences for each target gene were listed in Table 1, and relative expression levels were calculated using the 2^−ΔΔCT^ method.

**TABLE 1:** Primer sequence of target gene.

Primer	Forward	Reverse
CPD	5′-GCC​AAG​GGA​ACT​CGT​GGT​AA-3′	5′-AGC​CAG​TTG​ACA​TCA​TGC​GA-3′
GGCT	5′-ATC​TGC​ATG​GGT​GCG​AAA​GA-3′	5′-TGT​CTT​CCA​TTT​CGT​CGG​AGA-3′
SUZ12	5′-AGC​ATC​AAA​AGC​TTG​TCT​GCA​C-3′	5′-GGA​AAC​TGC​CAG​GGA​TGG​AA-3′
ZMYM2	5′-AGT​CTC​ACG​AAC​GTA​GGA​AAT​TC-3′	5′-GCA​ACC​AAA​GGT​GCA​GAA​GAT-3′
M-GAPDH	5′-TGT​GTC​CGT​CGT​GGA​TCT​GA-3′	5′-GAG​TTG​CTG​TTG​AAG​TCG​CA-3′

#### 2.7.6 Statistical analysis

GraphPad Prism 9.4.0 software was used for statistical analysis with one-way ANOVA.A value of *P* < 0.05 was considered significant, and data are presented as mean ± S.E.M. A statement to confirm that all methods were carried out in accordance with relevant guidelines and regulations.

## 3 Results

### 3.1 Identification of DEGs

**FIGURE 1:**
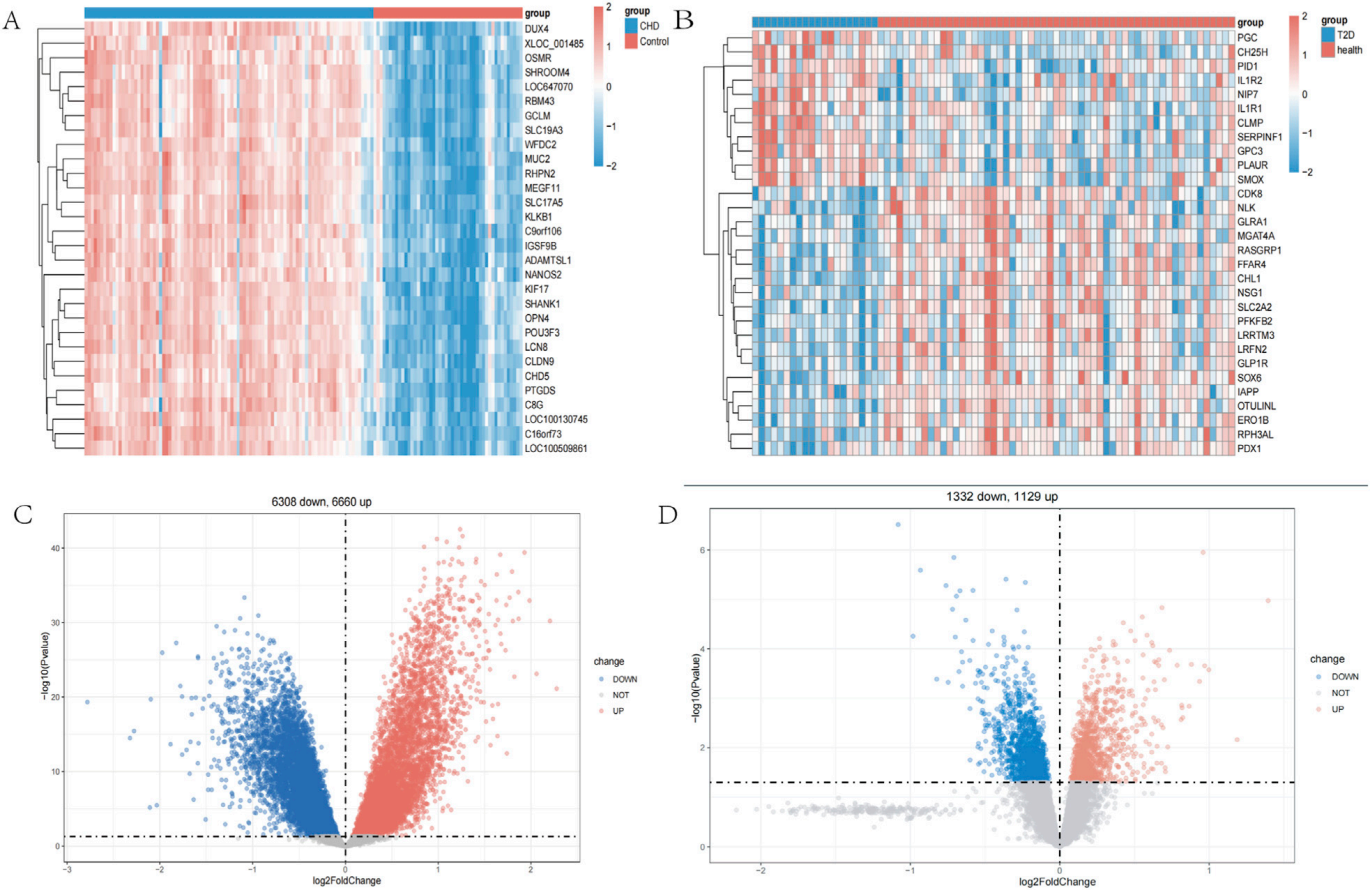
Identification of differentially expressed genes. **(A)** A heatmap of the top 30 DEGs in GSE113079. **(B)** A heatmap of the top 30 DEGs in GSE41762. **(C)** A volcano plot of DEGs in GSE113079. **(D)** A volcano plot of DEGs in GSE41762.

In the CHD dataset GSE113079, a total of 12,968 DEGs were identified, including 6,660 upregulated DEGs and 6,308 downregulated DEGs. In the T2D dataset GSE41762, a total of 52,461 DEGs were identified, including 11,129 upregulated DEGs and 13,332 downregulated DEGs. The heatmaps (Figures 1A,B) display the top 30 most significant DEGs for both diseases, while the volcano plots (Figures 1C,D) illustrate the expression patterns of the DEGs in both diseases.

### 3.2 WGCNA network construction and module identification

**FIGURE 2:**
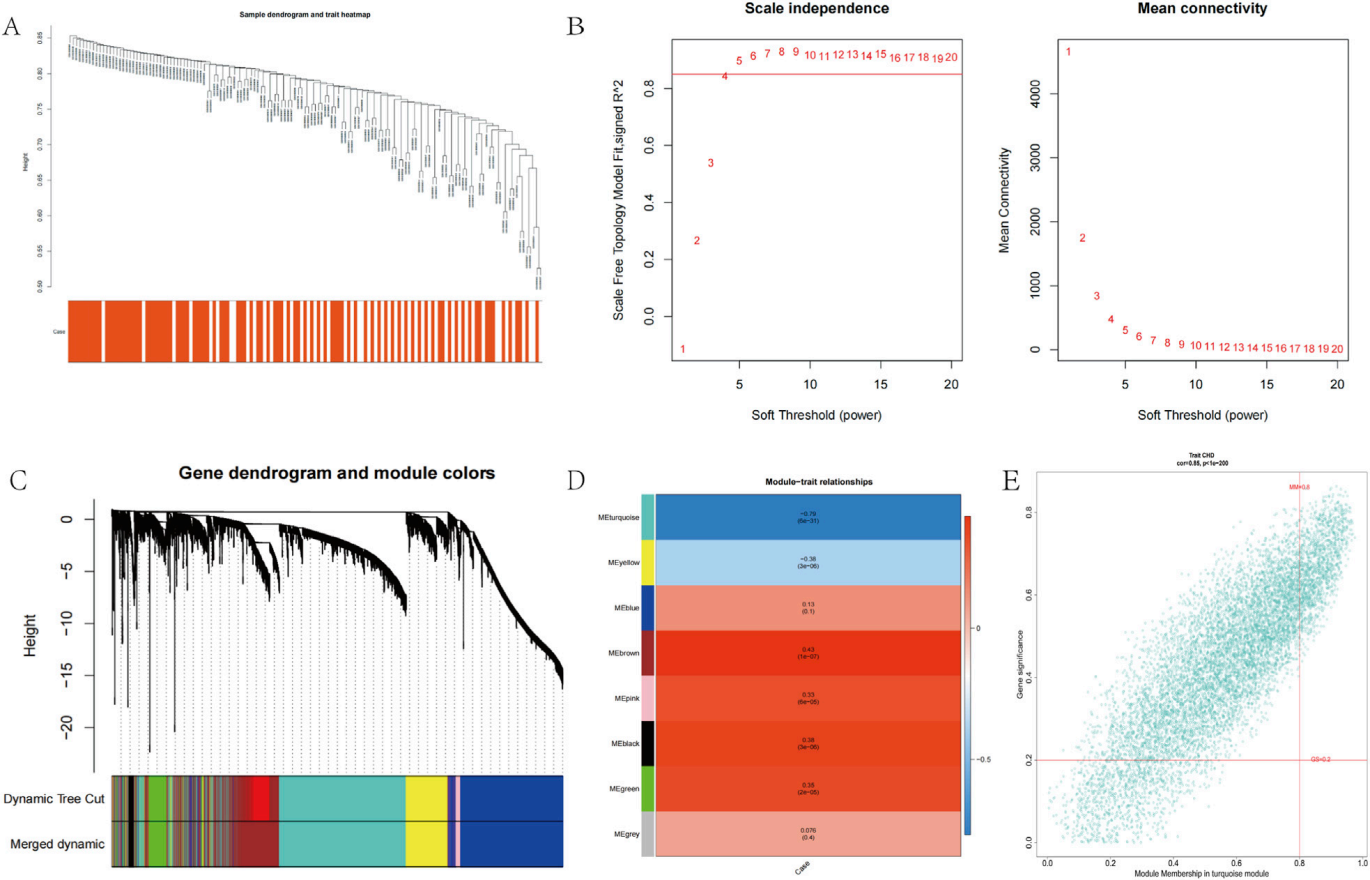
Co-expression analysis for differentially expressed genes. **(A)** Sample dendrogram and trait heatmap in GSE113079. **(B)** Scale-Free soft threshold selection. **(C)** Gene dendrogram and module colors. **(D)** Heatmap of the module-trait relationships. **(E)** Scatter plots of the degree and P-value of Cox regression in MEturquoise module.

No outliers were detected in the 141 samples from the GSE113079 dataset (Figure 2A). To construct a scale-free network (*R* ^*2*^ = 0.85), we set the soft-thresholding power β to 5 (Figure 2B). Using a hybrid dynamic tree-cutting algorithm with a minimum module size of 100 genes, 10 co-expression modules were initially identified, including the gray module containing unassigned genes (Figure 2C). Subsequently, modules exhibiting high similarity (with a module eigengene dissimilarity threshold, MEDissThres = 0.2) were merged, resulting in a final set of 8 distinct modules (Figure 2C). Pearson correlation analysis revealed that the MEturquoise module showed the strongest significant association with the CHD phenotype (cor = −0.79, P = 6e-31) (Figure 2D). Furthermore, within the MEturquoise module, MM and gene GS for CHD displayed a remarkably strong correlation (cor = 0.85, P = 1e-200) (Figure 2E). Based on these findings, the MEturquoise module was designated as the module of interest (termed the CHD module), and the 6,914 genes it contained were defined as CHD module genes.

### 3.3 Screening and enrichment analysis of shared genes

**FIGURE 3:**
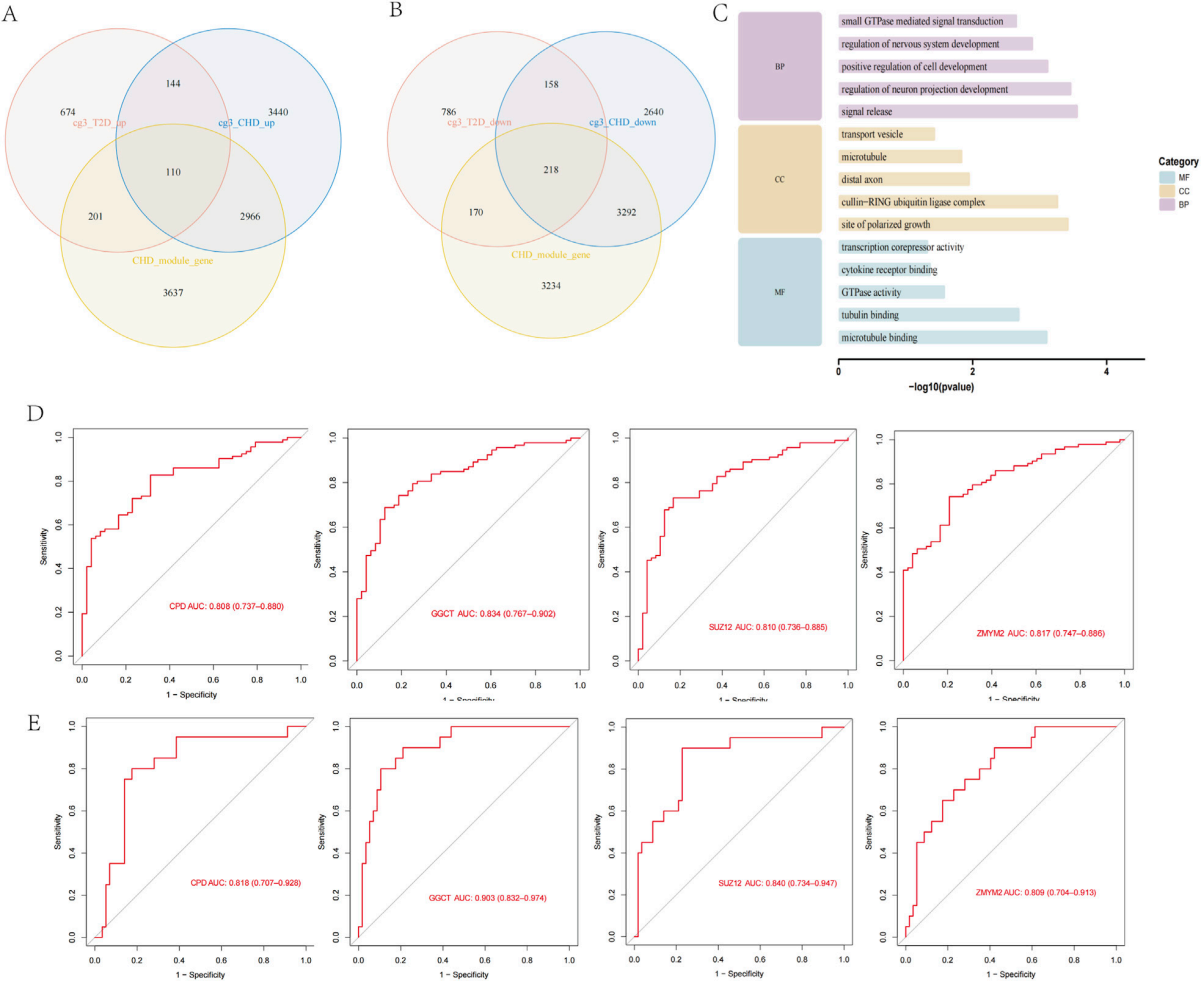
Screening of shared genes and identification of diagnostic biomarkers. **(A)** Screening of upregulated shared genes via Venn diagram. **(B)** Screening of downregulated shared genes via Venn diagram. **(C)** GO enrichment analysis of shared genes. **(D)** ROC analysis of diagnostic genes in CHD. **(E)** ROC analysis of diagnostic genes in T2D.

We intersected the 12,968 CHD DEGs, 6,914 CHD MEturquoise module genes, and 2,461 diabetes DEGs to identify shared genes between both diseases. The results revealed 110 overlapping genes in the upregulated set (Figure 3A) and 218 overlapping genes in the downregulated set (Figure 3B), with expression patterns of these 328 shared genes visualized in a heatmap across both disease cohorts (Supplementary Figure S1). We performed GO enrichment analysis on the 328 shared genes for both diseases to explore common regulatory pathways. The GO analysis indicated that these shared genes may be associated with specific biological processes such as positive regulation of nervous system development, positive regulation of neurogenesis, and positive regulation of cellular development (Figure 3C). We evaluated the diagnostic potential of the 328 shared genes by performing ROC curve analysis to assess their ability to discriminate between control and CHD samples, as well as between control and T2D samples. Genes with AUC values > 0.8 in both diseases were considered diagnostically significant. This analysis identified four genes (CPD, GGCT, SUZ12, ZMYM2) meeting this threshold, which were subsequently designated as diagnostic biomarkers (Figures 3D,E).

### 3.4 Prediction and validation of transcription factors

**FIGURE 4:**
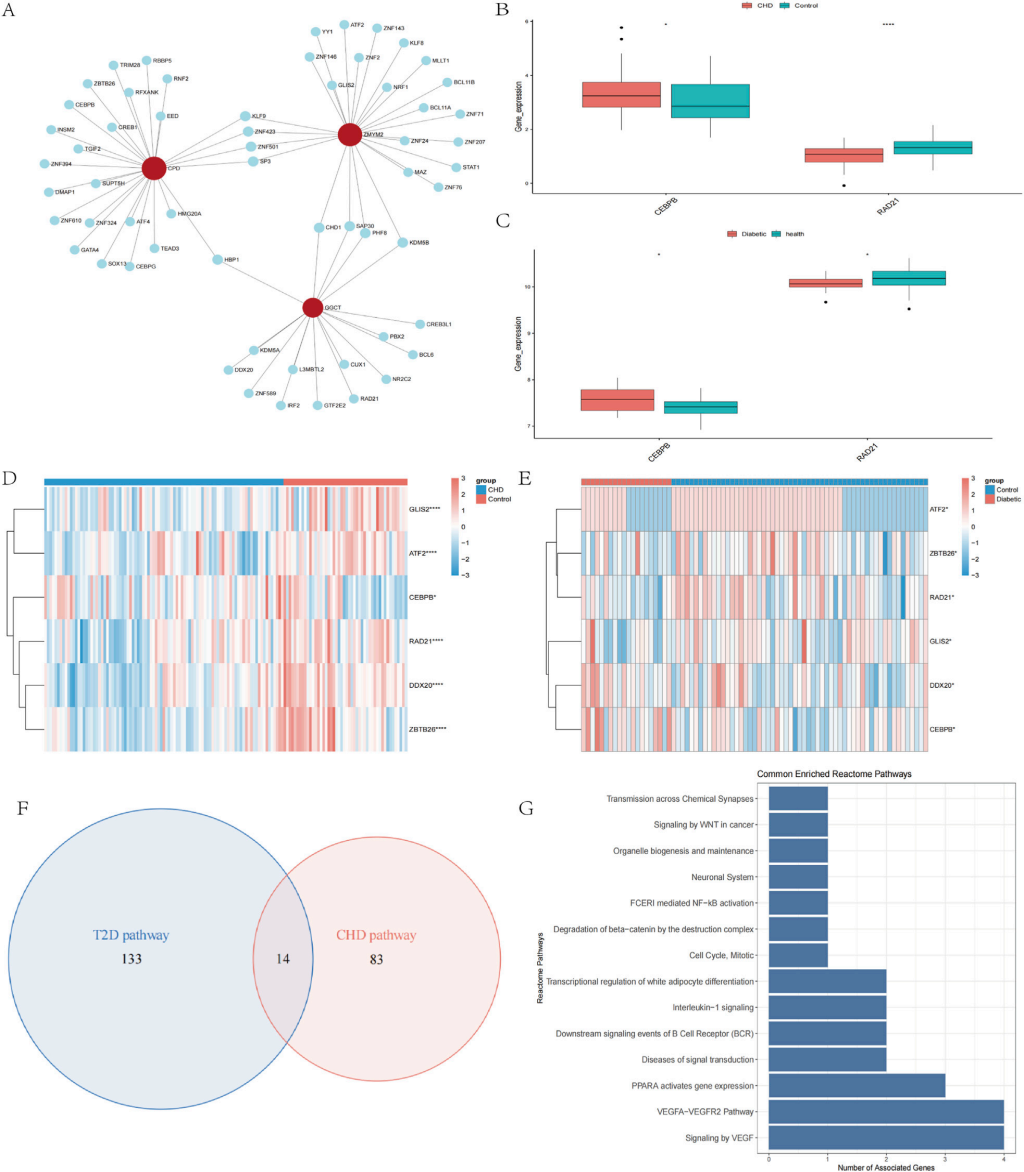
Regulatory networks and functional pathways of cross-disease biomarkers. **(A)** Prediction of TFs for Diagnostic Biomarkers. **(B,C)** Box plots of transcription factors CEBPB and RAD21 expression in CHD, T2D and normal controls. **(D,E)** Heatmap of TFs expression in CHD, T2D, and normal controls. **(F)** Venn diagram screening of co-enriched pathways in CHD and T2D. **(G)** Visualization of 14 co-enriched pathways. *P < 0.05, **P < 0.01, ***P < 0.001.

To elucidate the transcriptional regulatory network of the diagnostic genes, we employed Network Analyst (https://www.networkanalyst.ca) to predict associated TFs using screening thresholds of binding intensity <500 and interaction score <1. The regulatory network was constructed using Cytoscape, comprising 71 nodes (3 diagnostic genes and 67 TFs) and 76 edges (Figure 4A). We then extracted expression profiles of these 67 TFs from CHD and T2D datasets for differential expression analysis between disease and control groups (Figures 4D,E). TFs exhibiting consistent differential expression patterns in both diseases were prioritized. Results identified 2 TFs with concordant dysregulation: CEBPB was significantly upregulated in disease groups of both disorders (Figure 4B), while RAD21 showed consistent downregulation (Figure 4C). These findings suggest that CEBPB and RAD21 may serve as common regulators of the diagnostic genes across both diseases.

### 3.5 Single-gene functional enrichment analysis of shared diagnostic biomarkers

To investigate the common pathways of the screened diagnostic biomarkers in CHD and T2D, we acquired pathway information from the GO (Supplementary Figure S2), KEGG (Supplementary Figure S3), and Reactome databases (Supplementary Figure S4) before conducting single-gene functional enrichment analysis for both diseases separately. Disease-related pathways were additionally retrieved from the Comparative Toxicogenomics Database (CTD). Intersection of the four biomarkers’ single-gene enrichment results with CTD-derived pathways demonstrated significant enrichment in leukocyte-mediated signaling pathways, Vascular Endothelial Growth Factor (VEGF) signal transduction, and B-cell receptor (BCR) downstream signaling events for the CHD group, while the T2D group primarily showed enrichment in β-cell development regulation, BCR downstream signaling, insulin secretion regulation, and insulin processing. Subsequent intersection analysis of both groups’ disease-specific pathways identified 14 shared pathways (Figure 4F) mainly involving signal transduction-related diseases, VEGF signaling pathways, and the VEGFA-VEGFR2 signaling axis (Figure 4G), indicating that the four shared diagnostic genes may interact through vascular dysfunction and metabolic dysregulation.

### 3.6 Establishment of mouse models for CHD and T2D

**FIGURE 5:**
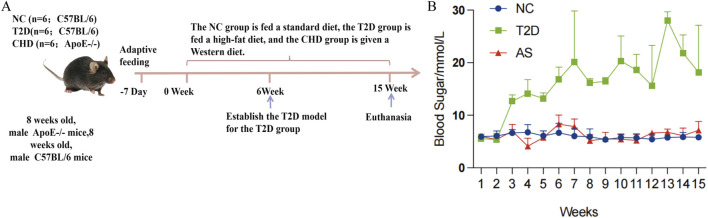
Development and validation of mouse disease models. **(A)** Flow chart of animal experiments. **(B)** Blood glucose monitoring in experimental mice.

**FIGURE 6:**
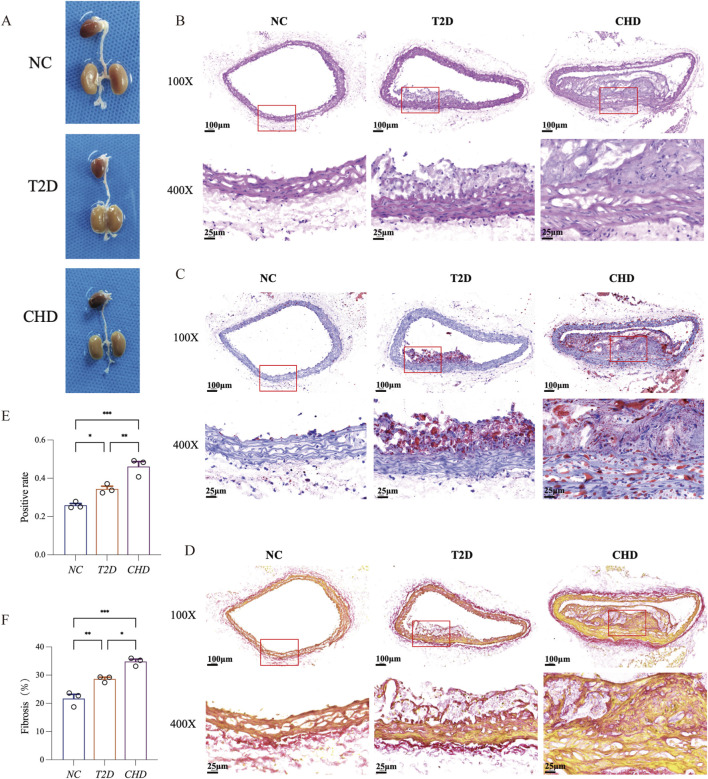
Comparative histopathology of aortic vessels in T2D, CHD and normal mice. **(A)** Representative aortic samples from CHD, T2D, and normal control mice. **(B)** H&E staining of mouse aortic sections. **(C)** Oil Red O staining of mouse aortic sections. **(D)** Sirius Red staining of mouse aortic sections. **(E)** Quantitative analysis of Oil Red O positive area in aortic lesions. **(F)** Quantitative analysis of Sirius Red positive area in aortic lesions (n = 3 per group). The data are represented as means ± s.e.m. *P < 0.05, **P < 0.01, ***P < 0.001.

To validate the expression profiles of the four diagnostic biomarkers in CHD and T2D,we conducted *in vivo* experiments using a standardized modeling protocol (Figure 5A): developed a modeling flowchart and dynamically monitored blood glucose levels (T2D group exhibited significantly elevated blood glucose after STZ induction, with two consecutive FBG measurements ≥11.1 mmol/L) (Figure 5B); harvested aortic tissues from control, CHD-model, and T2D-model mice post-modeling (Figure 6A), followed by histological assessments including HE staining for vascular pathology (Figure 6B), Oil Red O staining for quantitative lipid deposition (Figures 6C,E), and Sirius Red staining for collagen distribution (Figures 6D,F). Results demonstrated that the CHD-model group exhibited typical atherosclerotic plaques (lipid cores with collagen fiber hyperplasia), while the T2D-model group displayed thickened vascular basement membranes combined with lipid-collagen complex deposition, successfully recapitulating the core pathological phenotypes of both diseases.

### 3.7 Expression validation of shared diagnostic biomarkers

**FIGURE 7:**
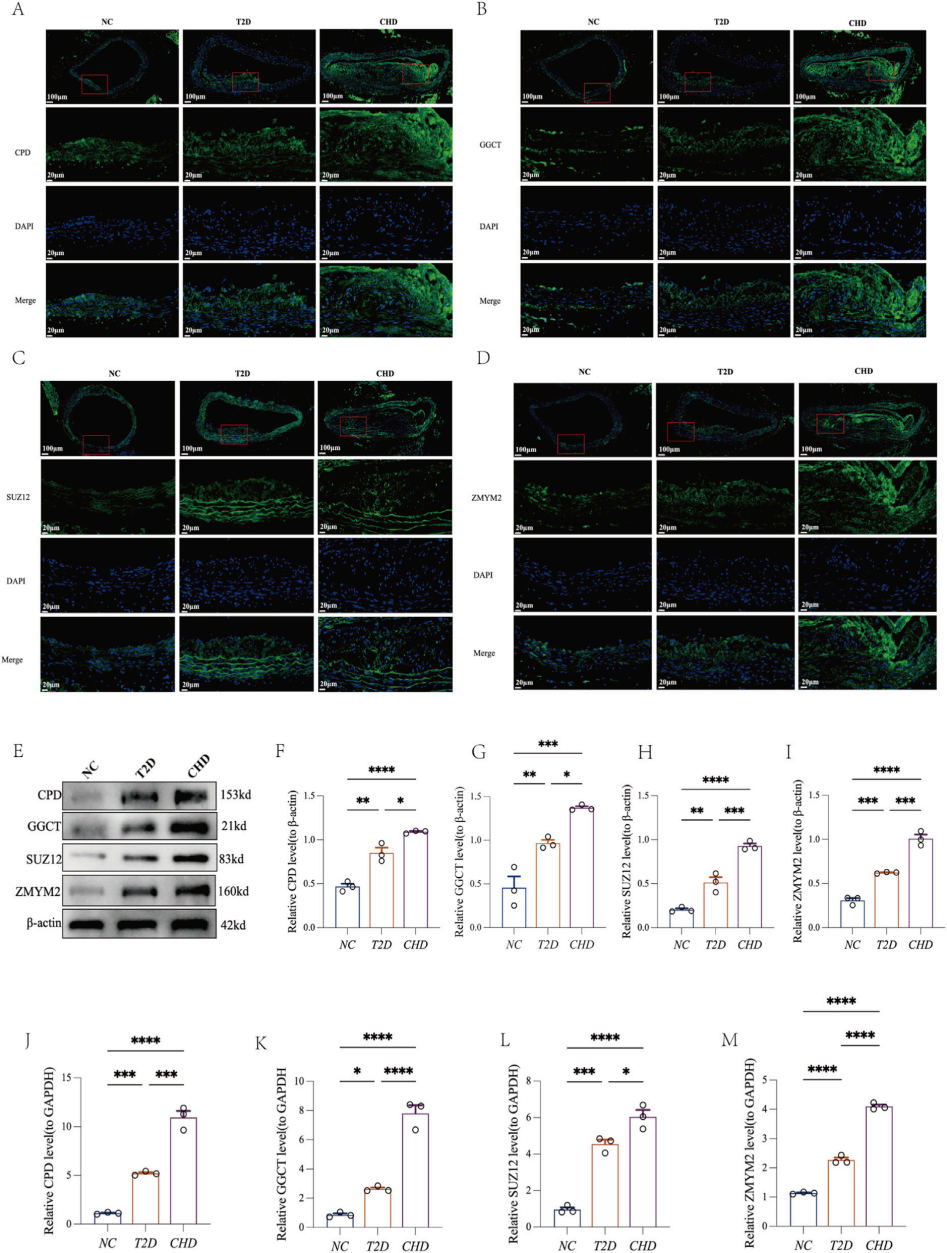
Four shared diagnostic biomarkers exhibit consistent upregulation in both CHD and T2D. **(A–D)** Immunofluorescence of CPD, GGCT, SUZ12, and ZMYM2 in mouse aortic tissues. **(E–I)** Representative Western blot of CPD, GGCT, SUZ12, ZMYM2, and β-actin in mouse aortic tissues with quantification analysis (n = 3 per group). **(J–M)** RT-qPCR analysis of CPD, GGCT, SUZ12, and ZMYM2 in mouse aortic tissues (n = 3 per group). The data are represented as means ± s.e.m. *P < 0.05, **P < 0.01, ***P < 0.001.

To delineate the expression profiles of shared diagnostic biomarkers (CPD, GGCT, SUZ12, ZMYM2) in mouse models of CHD and T2D, we performed multi-platform validation on aortic tissues using immunofluorescence (Figures 7A-D), Western blotting (Figures 7E-I), and RT-qPCR (Figures 7J-M). Results demonstrated that compared with the normal control group (NC group), CPD, GGCT, SUZ12, and ZMYM2 exhibited significantly elevated expression in both CHD and T2D model groups (*P* < 0.05), with consistent upregulation trends across disease models. These findings indicate that these four genes may serve as shared diagnostic biomarkers for CHD-T2D comorbidity.

## 4 Discussion

Our study aims to preliminarily explore the potential correlation and molecular mechanisms between CHD and T2D, identify core genes and biological processes, and provide background for future research. Firstly, by integrating and analyzing transcriptomic data from CHD and T2D datasets in the GEO database, we screened for DEGs associated with each disease. Secondly, we applied WGCNA to identify feature genes within network modules and intersected these with the DEGs. Through this three-way intersection, we obtained a set of shared genes for both diseases. Thirdly, we performed GO enrichment analysis on these shared genes. The results revealed that these genes are primarily associated with the regulation of nervous system development, K48-linked protein ubiquitination processes, and cullin-RING ubiquitin ligase complexes, among other functions. Fourthly, based on ROC curve analysis (AUC >0.8), we further screened and identified four cross-disease diagnostic biomarkers: CPD, GGCT, SUZ12, and ZMYM2. Concurrently, we constructed their TF regulatory network. Analysis of this network revealed consistent expression trends for the TFs CEBPB (co-upregulated in both diseases) and RAD21 (co-downregulated in both diseases). Fifthly, using single-gene GSEA combined with pathway cross-validation from the Comparative Toxicogenomics Database, we identified shared pathways between the two diseases (e.g., VEGF signaling, BCR downstream signaling events, cell cycle regulation, Wingless-related Integration Site (WNT) signaling in cancer). These pathways suggest potential molecular mechanisms through which the core genes may commonly regulate both CHD and T2D. Finally, through the construction of CHD/T2D mouse models combined with immunofluorescence, RT-qPCR, and Western blotting, we successfully validated the differential expression of the four diagnostic biomarkers in both disease models.

Carboxypeptidase D (CPD), a membrane-bound metallocarboxypeptidase primarily localized to the trans-Golgi network, processes peptides/prohormones by cleaving C-terminal arginine or lysine residues from protein substrates (Song and Fricker, 1995; Tan et al., 1997; Song and Fricker, 1996). It plays significant roles in inflammation and cancer pathology. Interferon-γ and lipopolysaccharide induce its expression in macrophages, stimulating nitric oxide (NO) production (Hadkar and Skidgel, 2001); a pleiotropic signaling molecule critical for physiology but also implicated in promoting tumor growth, invasion, and angiogenesis (Thomsen and Miles, 1998; Orucevic et al., 1999; Jadeski and Lala, 1999). In breast (Abdelmagid and Too, 2008) and prostate cancers, CPD is upregulated by hormones including prolactin, estrogen, and testosterone, driving oncogenic effects through promoting NO generation and enhancing tumor cell survival (Thomas et al., 2012). Furthermore, studies confirm that cytokine-induced impairment of islet function is mediated by NO. In IL-1β-treated isolated rat/human islets or Fluorescence-Activated Cell Sorting (FACS)-purified β cells, the Nitric Oxide Synthase (NOS) inhibitor N(G)-Monomethyl-L-arginine (L-NMMA) blocks glucose-stimulated insulin secretion inhibition, effectively preventing insulin secretory failure (Corbett et al., 1991; Corbett et al., 1993; Corbett et al., 1992). Immune response-generated NO exhibits cytotoxicity, damaging adjacent tissues and producer cells, leading to local tissue destruction, pathological vasodilation, and vascular permeability alterations. Studies have shown that CPD, as a potential candidate gene for T2D, may contribute to the development of the diabetic phenotype by modulating multiple diabetes-related signaling pathways. These include key biological processes such as adipocytokine signaling, glycerolipid metabolism, PPAR signaling, T cell receptor signaling, and insulin signaling pathways (Liu et al., 2015). Notably, research directly investigating CPD’s mechanistic role in T2D and CHD remains critically insufficient, necessitating in-depth exploration of CPD’s regulatory functions in the pathological processes of these chronic diseases. In this study, the upregulation of CPD in the T2D mouse model was accompanied by persistent hyperglycemia and vascular inflammation, suggesting that inhibition of CPD activity may hold potential therapeutic value. For example, developing CPD-specific inhibitors to reduce excessive NO production could not only alleviate vascular basement membrane thickening associated with T2D but also decrease plaque inflammation in CHD, thereby achieving dual protection for both metabolic and vascular systems.

γ-Glutamyl cyclotransferase (GGCT) (encoded by the C7orf24gene) serves as a core enzyme in glutathione metabolism, catalyzing the conversion of γ-glutamyl peptides into5-oxoproline and free amino acids (Kageyama et al., 2015). Our study reveals that GGCT is a key shared gene between CHD and T2D, exhibiting an upregulation trend in both conditions. This gene demonstrates aberrant accumulation in multiple cancers, including breast, ovarian, cervical, lung, bladder, and prostate cancers, as well as colorectal carcinoma, osteosarcoma, and glioma (Amano et al., 2012; Kageyama et al., 2004; Takemura et al., 2014). It drives tumor progression by promoting cancer cell proliferation, invasion, migration, epithelial-mesenchymal transition (EMT), and glycolysis (Taniguchi et al., 2022; Li et al., 2018; Xu et al., 2020). Studies have shown that GGCT may influence the proliferation and migration of papillary thyroid carcinoma (PTC) cells by regulating the MAPK/ERK signaling pathway (Zhang et al., 2022). In addition, GGCT may also be involved in the regulation of glutamate metabolism–related pathways, which are associated with the interconnection between T2D and coronary artery disease (CAD) (Giuffrida et al., 2024). This finding suggests that the potential application of GGCT inhibitors in the treatment of comorbid conditions warrants further exploration—by restoring glutathione levels, alleviating oxidative damage in vascular cells, and improving pancreatic β-cell function in T2D patients, given that oxidative stress is a major cause of β-cell apoptosis (Dinić et al., 2022).

SUZ12 (Suppressor of zeste 12 Homolog) is a core component of the Polycomb Repressive Complex 2 (PRC2) histone methyltransferase (HMTase) complex. This complex catalyzes the trimethylation of histone H3 at lysine 27 (H3K27me3), thereby mediating gene silencing and playing a critical role in stem cell maintenance and development (Vizán et al., 2015; Lee et al., 2015; Teng et al., 2023). While typically difficult to detect in normal tissues, SUZ12 has been found to be amplified and overexpressed in various human cancers, such as ovarian cancer, mantle cell lymphoma, breast cancer, head and neck squamous cell carcinoma (HNSCC), colorectal cancer, and non-small cell lung cancer (Li et al., 2012; Iliopoulos et al., 2010; Martín-Pérez et al., 2010). This study found that SUZ12 expression is upregulated in both CHD and T2D, revealing that SUZ12 may be involved in non-cancer diseases. Given that SUZ12 is a core regulator of PRC2-mediated gene silencing, its dysregulation in CHD/T2D suggests that epigenetic dysregulation could be a significant factor in the pathogenesis and progression of these diseases. Future studies are urgently needed to explore the specific functions of SUZ12 in the cardiovascular and metabolic systems, the mechanisms regulating its expression, and its translational clinical value as a potential therapeutic target for CHD/T2D.In this context, translating these mechanistic insights into clinical applications could hold substantial diagnostic and preventive value. For example, for patients with T2D (a high-risk population for CHD), a diagnostic kit detecting SUZ12 expression in peripheral blood mononuclear cells could be developed. If the test result indicates high SUZ12 expression, it may suggest that the patient has entered a “pre-vascular lesion stage” (before the onset of obvious symptoms such as chest tightness or chest pain). At this stage, early intervention—such as intensified lipid-lowering and anti-inflammatory therapies—should be initiated to reduce the risk of acute coronary events.

ZMYM2 (also known as ZNF198, FIM, or RAMP) is a core member of the MYM-type zinc finger protein family. The 150 kDa nuclear transcription factor it encodes (containing 1377 amino acids) localizes to promyelocytic leukemia (PML) nuclear bodies and possesses DNA/RNA binding and protein interaction functions (Mackay and Crossley, 1998; Still and Cowell, 1998; Gocke and Yu, 2008). At the molecular mechanism level, ZMYM2 mutations lead to congenital anomalies of the kidney and urinary tract (CAKUT)-like defects (Dai et al., 2025). CAKUT is inherently a multisystem phenotype, encompassing progressive decline in renal function, pancreatic abnormalities, diabetes, and neurological deficits (Boeri et al., 2024). In CAKUT patients carrying heterozygous nonsense or frameshift mutations in ZMYM2, various neurological manifestations have been observed, including microcephaly, developmental delay, intellectual disability, speech delay, and infantile hypotonia (Connaughton et al., 2020; Jourdeuil et al., 2025). Furthermore, male carriers appear to be more susceptible to synergistic influences such as genetic predisposition, genitourinary tract infections, environmental pollutants, and smoking (Connaughton et al., 2020). Significantly, the ZMYM2 heterozygous mutant mouse model also exhibits metabolic abnormalities, including chronic mild hyperglycemia (progressively elevated blood glucose levels until 90 days of age), significantly reduced fasting serum insulin, and impaired insulin secretion capacity (Clissold et al., 2015; Pu et al., 2025). The ZMYM2–FGFR1 fusion gene has been reported to drive the development and progression of T-cell lymphoma through sustained activation of the Notch1 signaling pathway (Ren and Cowell, 2011). In this study, ZMYM2 was found to be upregulated in the vascular tissues of comorbid patients, suggesting that it may serve as a critical node linking metabolic dysregulation and vascular injury. Future studies could explore the downstream regulatory factors of ZMYM2—such as the transcription factor CEBPB predicted in this study—and investigate whether modulation of the ZMYM2–CEBPB axis can simultaneously improve glucose metabolism in T2D and vascular inflammation in CHD, thereby achieving synergistic therapeutic effects for the comorbidity.

TF network analysis identified CEBPB and RAD21 as common regulators in both CHD and T2D. CCAAT/Enhancer-Binding Protein beta (CEBPB), a key member of the CCAAT/Enhancer Binding Protein (C/EBP) family, is essential for cell growth, differentiation, and cardiovascular remodeling, particularly in physiological hypertrophy and heart failure (Boström et al., 2010). Elevated CEBPB levels are linked to vascular remodeling and atherosclerosis, suggesting it could be a therapeutic target for cardiovascular diseases like vascular inflammation (Takata et al., 2002). CEBPB also plays a role in adipocyte metabolism, regulating plasma free fatty acids and insulin signaling in skeletal muscle, and is elevated in insulin-resistant obese individuals, indicating its role in metabolic processes (Wang et al., 2000)). Therefore, CEBPB may be the key transcriptional regulator mediating the shared pathophysiological processes of these two diseases, particularly chronic inflammation, metabolic dysregulation, and tissue remodeling. RAD21, a DNA double-strand break repair protein, is a subunit of the chromosome cohesion complex, crucial for gene transcription, nuclear structure, apoptosis, and hematopoietic development. It has been found to be abnormally expressed in various cancers, including colorectal, endometrial, and breast cancers, with implications for tumor development, prognosis, and treatment (Jiao et al., 2023). However, its role in the onset and progression of T2D and CHD remains unexplored. To date, no studies have reported direct interactions between CEBPB, RAD21, and the four core target genes. In this study, these transcription factors were identified as potential regulators based on bioinformatic predictions from the NetworkAnalyst database. Future validation using ChIP-qPCR and luciferase reporter assays is required to confirm their direct binding relationships with the target genes.

This study suggests that the shared diagnostic biomarkers between CHD and T2D are associated with the VEGF signaling pathway. VEGF, a key pro-angiogenic factor, critically regulates vascular permeability across various diseases (Phoenix et al., 2022). In diabetic models, upregulated VEGF-B binds to its receptor VEGFR1, promoting fatty acid uptake in muscle and cardiac tissue through FATP3/FATP4. This drives lipid deposition, insulin resistance, and secondary hyperglycemia (Hagberg et al., 2010). Inhibition of the VEGF-B signaling pathway improves glucose tolerance and insulin resistance (Robciuc et al., 2016). During atherosclerosis progression, high VEGF expression increases plaque vascular density, facilitating the growth of pathological neovessels into the intima and thereby accelerating disease progression. Furthermore, in CHD patients, myocardial tissue oversecretes VEGF-A in response to local inflammation, mechanical stretch, and cytokine stimulation, leading to myocardial fibrotic remodeling, contractile dysfunction, and impaired repair capacity (Garcia et al., 2019). Mechanistically, loss of SUZ12 function has been reported to promote angiogenesis and tumor progression in T-cell acute lymphoblastic leukemia (T-ALL) through modulation of the VEGF signaling pathway (Broux et al., 2019). The study found that CPD is upregulated in breast cancer through prolactin and androgen signaling, which promotes NO production. The generated NO subsequently induces VEGF-C expression, thereby enhancing tumor angiogenesis and progression. Thus, CPD and VEGF exhibit a positive regulatory relationship and jointly contribute to the malignant transformation of breast cancer (Thomas et al., 2017). Therefore, VEGF likely plays a central role in the vicious cycle of CHD-T2D comorbidity by coordinating abnormal vascular permeability (exacerbating lipid infiltration/inflammation), pathological neovascularization, and dysregulated fatty acid transport.

This study innovatively integrated bioinformatics analysis with animal experimental validation, identifying CPD, GGCT, SUZ12, and ZMYM2 as shared diagnostic biomarkers for CHD and T2D. It further constructed the transcription factor regulatory networks of these biomarkers across both diseases, offering a unified molecular diagnostic framework and elucidating potential synergistic pathological mechanisms for the comorbidity. This study has several limitations. First, although the associations between biomarkers and diseases were explored, their causal relationships and regulatory mechanisms with transcription factors have not been fully validated. Future studies will employ CRISPR technology or gene knockout mouse models to investigate these interactions in greater depth. Second, the current sample size is relatively small and the mechanistic exploration is limited, which may introduce bias; expanding the sample size in future studies will improve representativeness and statistical power. Third, as the study relied primarily on mouse models and lacked validation in human samples, the clinical translatability is restricted. Subsequent work will examine the expression of key genes identified in mouse models within human samples to enhance clinical relevance. In addition, the absence of *in vitro* functional assays is another limitation; future studies will incorporate knockdown/overexpression experiments in relevant cell lines to further validate the findings. Finally, due to limitations in sample availability, extensive clinical data analysis could not be performed. Future multi-center collaborative studies will be conducted to provide stronger clinical support for the conclusions. Nevertheless, this pioneering work has systematically revealed shared diagnostic biomarkers for CHD-T2D comorbidity, laying a crucial foundation for the precise diagnosis and treatment of this comorbidity.

## 5 Conclusion

This study identified CPD, GGCT, SUZ12, and ZMYM2 as core shared diagnostic biomarkers of T2D–CHD comorbidity. These biomarkers were significantly upregulated in the diseased vasculature of both conditions, with transcription factors such as CEBPB and RAD21 regulating their expression, and pathways such as VEGF participating in the pathological mechanisms of the comorbidity. This finding provides new targets for the diagnosis and mechanistic study of the comorbidity. However, the study is limited by the sample types and the insufficient depth of mechanistic exploration. Future work should expand clinical sample validation and employ gene-editing models to clarify the causal roles of these biomarkers, further elucidating their mechanistic functions within the VEGF pathway, thereby advancing precision diagnosis and treatment of the comorbidity.

## Data Availability

Publicly available datasets were analyzed in this study. This data can be found here: Gene Expression Omnibus database, GSE113079 and GSE41762.

## Supplementary material

SUPPLEMENTARY FIGURE S1 Heatmaps of Shared Genes in CHD and T2D. **(A)** Heatmap of shared genes in CHD. **(B)** Heatmap of shared genes in T2D.

SUPPLEMENTARY FIGURE S2 GO enrichment analysis of shared diagnostic biomarkers for CHD and T2D. **(A–D)** Top 10 Enriched GO pathways for single-gene in CHD. **(E–H)** Top 10 enriched GO pathways for single-gene in T2D.

SUPPLEMENTARY FIGURE S3 KEGG enrichment analysis of shared diagnostic biomarkers for CHD and T2D. **(A–D)** Top 10 enriched KEGG pathways for single-gene in CHD. **(E–H)** Top 10 enriched KEGG pathways for single-gene in T2D.

SUPPLEMENTARY FIGURE S4 Reactome enrichment analysis of shared diagnostic biomarkers for CHD and T2D. **(A–D)** Top 10 enriched reactome pathways for single genes in CHD. **(E–H)** Top 10 enriched reactome pathways for single genes in T2D.
