# Supplementary figures and images for: Computational-experimental strategy identifies Co-upregulated biomarkers linking coronary heart disease and type 2 diabetes pathogenesis
Source: Front Genet. 2025 Nov 12;16:1673303. doi: 10.3389/fgene.2025.1673303 (PMC12646545; doi:10.3389/fgene.2025.1673303)

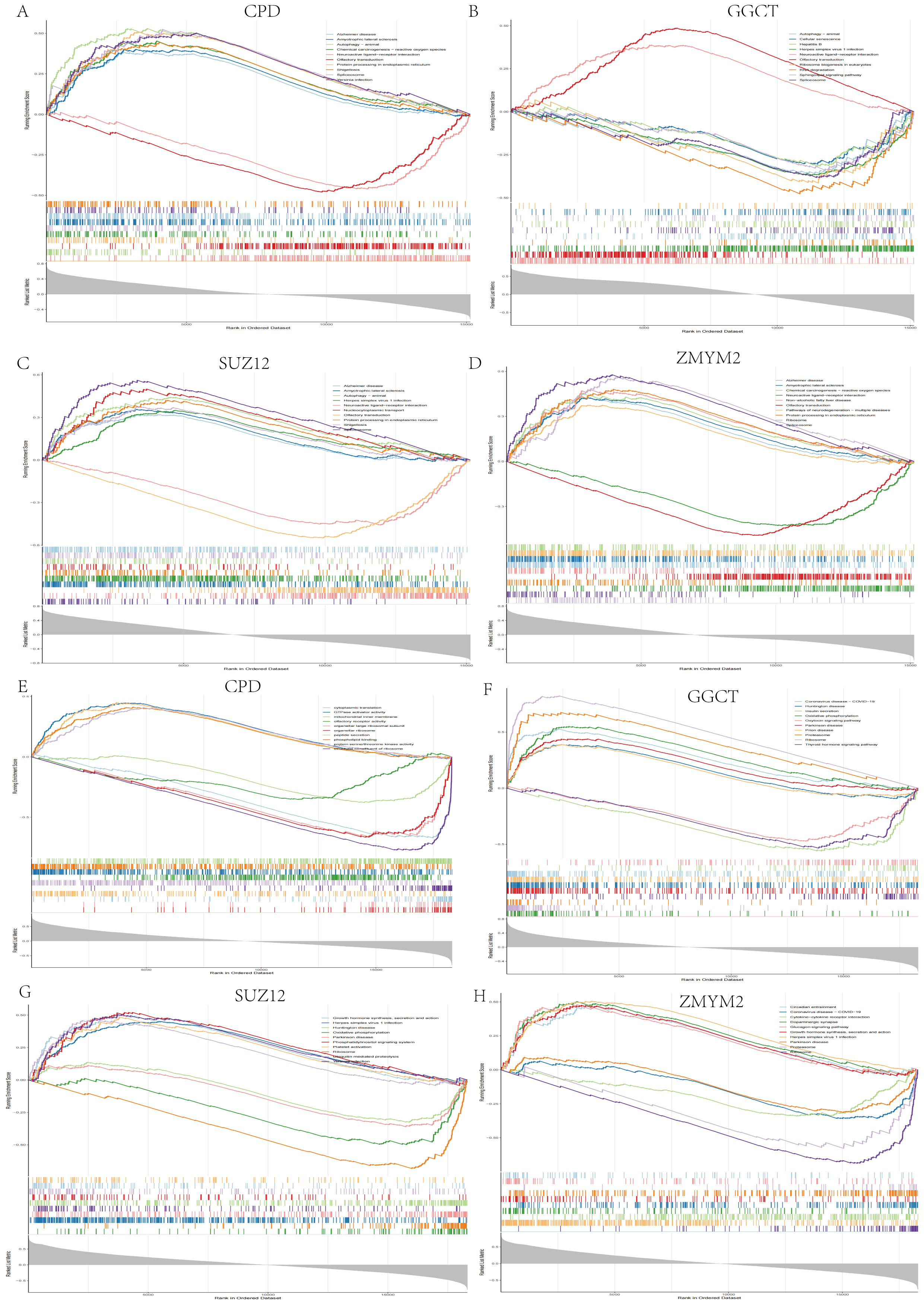

Supplement: Supplementary file 1 [file Image3.tif]

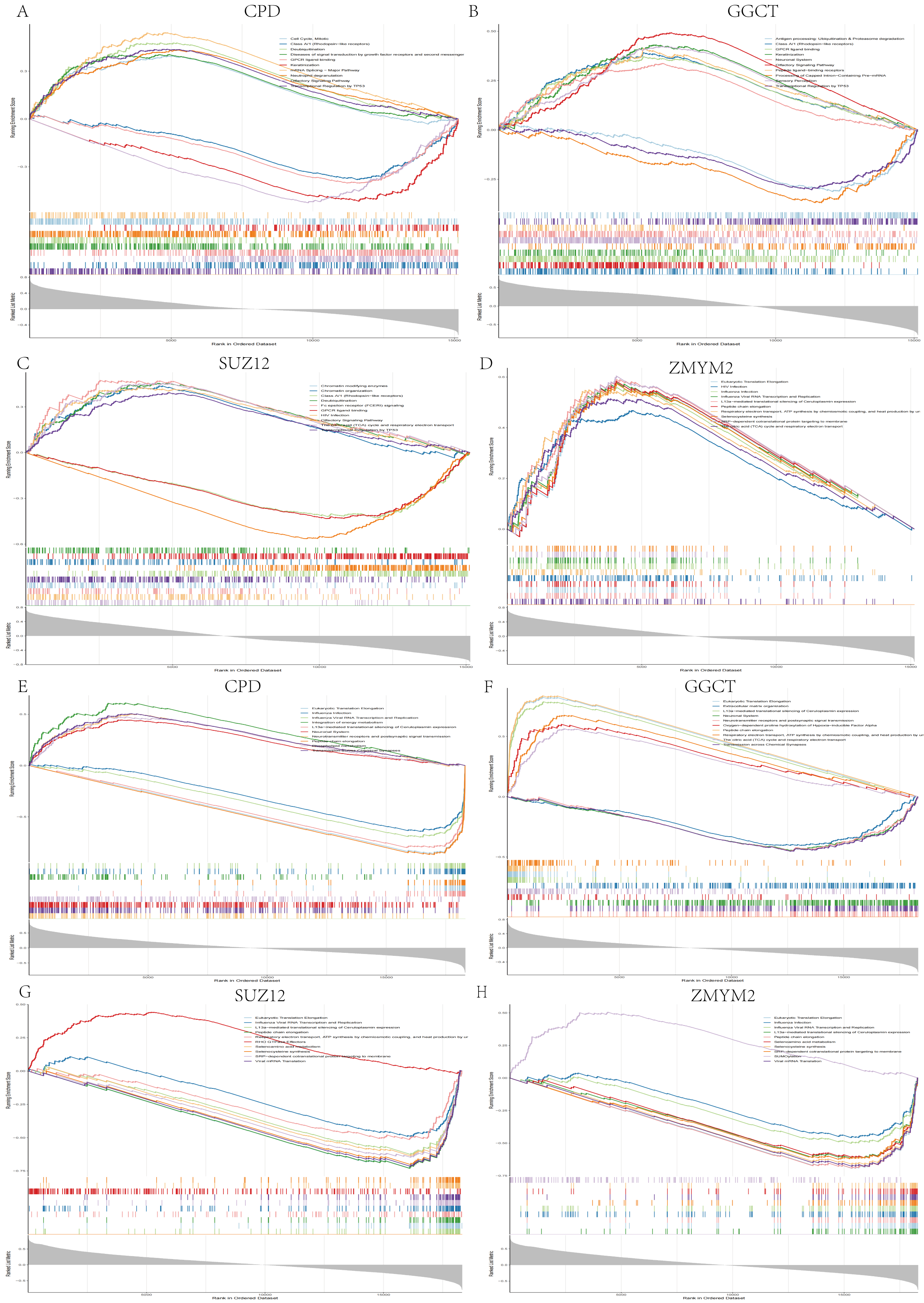

Supplement: Supplementary file 2 [file Image4.tif]

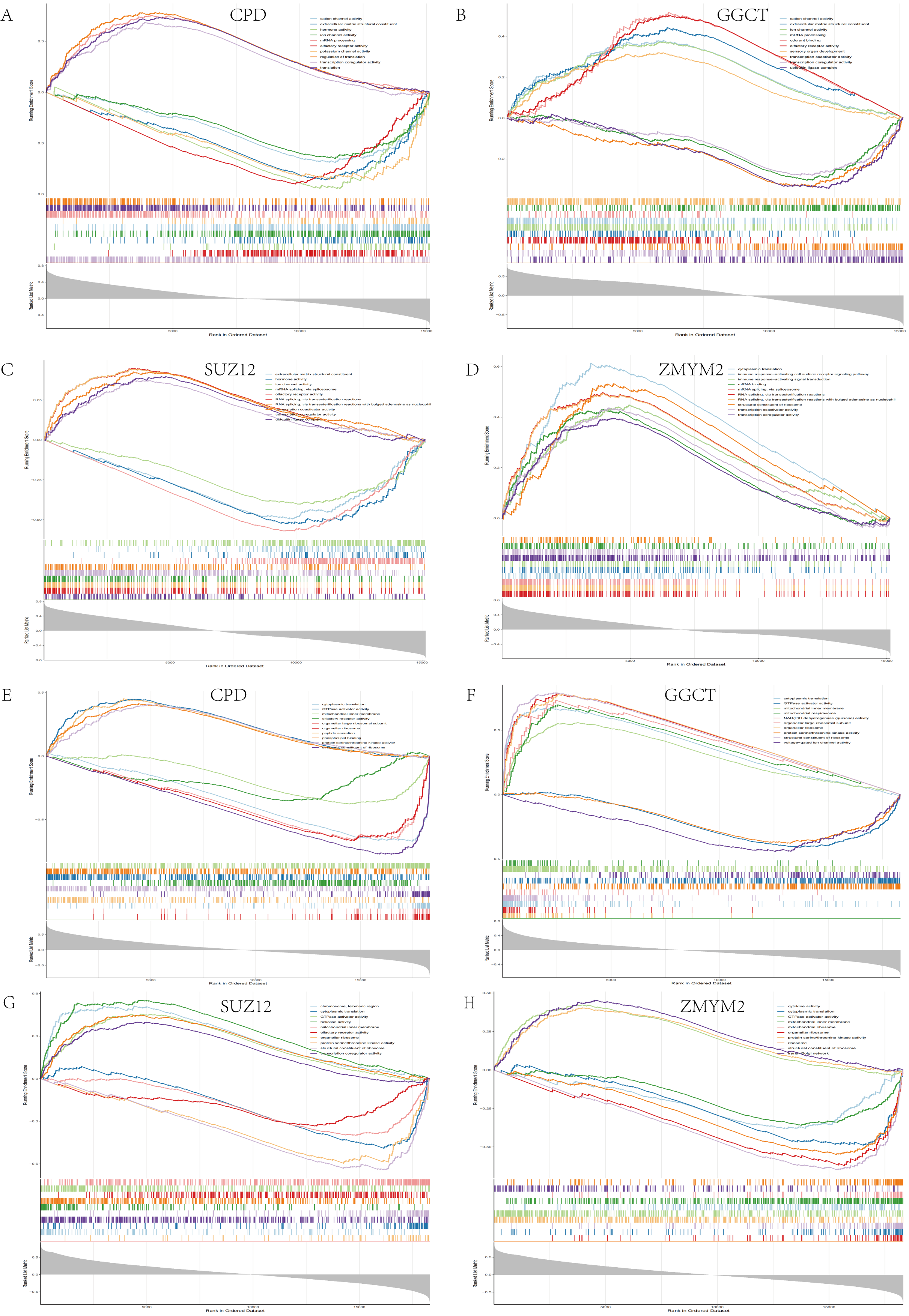

Supplement: Supplementary file 3 [file Image2.tif]

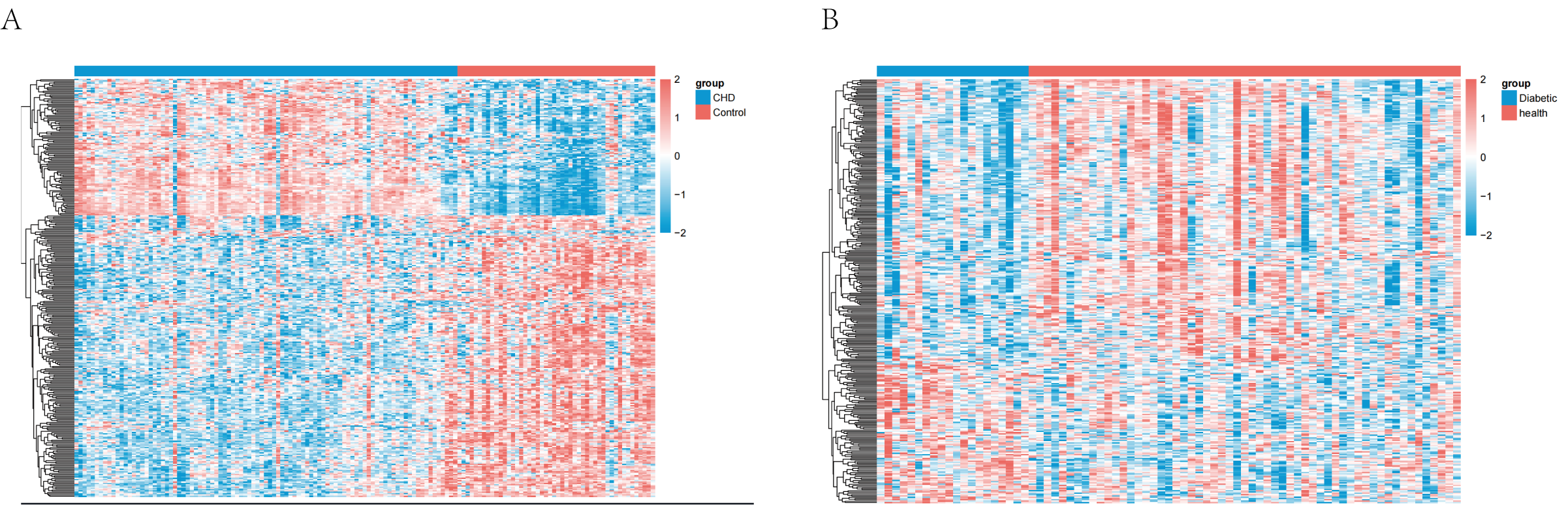

Supplement: Supplementary file 4 [file Image1.tif]
